# Pulsed field ablation for pulmonary vein and left atrial posterior wall isolation in persistent atrial fibrillation: a narrative review

**DOI:** 10.1097/MS9.0000000000004219

**Published:** 2025-10-29

**Authors:** Tochukwu R. Nzeako, Chukwuka Elendu, Ebubechukwu Ezeh, Daniel E. Otobrise, Olayemi Adeniran, Samuel B. Governor, Ike Ifedili, Shoshanah Kahn

**Affiliations:** a Department of Cardiology, Christiana Care Hospital, Delaware, USA; b Division of Medicine, Federal University Teaching Hospital, Owerri, Nigeria; c Department of Cardiology, University of Kansas Medical Center, Kansas City, Kansas, USA; d Division of Medicine, University of Medical Sciences, Ondo, Nigeria; e Department of Cardiovascular Diseases, SUNY Downstate University Hospital, Brooklyn, New York, USA; f Division of Medicine, St. John’s Episcopal Hospital, Far Rockaway, New York, USA; g Department of Cardiology, University of Tennessee Health Science Center, Memphis, Tennessee, USA; h Department of Cardiology, North Shore University Hospital, Long Island, New York, USA

**Keywords:** catheter ablation, FARAPULSE, left atrial posterior wall isolation, persistent atrial fibrillation, pulsed-field ablation

## Abstract

Pulsed-field ablation (PFA) is an emerging technique for managing atrial fibrillation (AF). Still, its role in persistent AF (PeAF) with left atrial posterior wall isolation (LAPWI) remains to be defined. The ATHENA registry evaluated 249 PeAF patients across 9 Italian centers using the FARAPULSE PFA system, with 57.6% undergoing LAPWI. Electrical isolation of the posterior wall was achieved in all cases, with a first-pass success rate of 88.8%. The procedure demonstrated a favorable safety profile, with no major periprocedural complications and only a low rate of minor events (2.4%). Secondary endpoints further assessed long-term efficacy and safety, with 16.5% of patients experiencing arrhythmic recurrence beyond the blanking period at a median follow-up of 273 days, corresponding to an overall freedom from recurrence of 83.5%. Complication rates remained low throughout follow-up, with no late-occurring major adverse events reported. Redo procedures were required in 15.3% of LAPWI patients compared with lower rates in the PVI-only group, suggesting a potential benefit of adjunctive posterior wall ablation. These findings support PFA as a feasible, safe, and efficient approach for LAPWI in PeAF, even with fluoroscopy-only guidance.

## Public summary

PFA is a novel technique for treating PeAF by isolating the pulmonary veins and the left atrial posterior wall. Unlike traditional thermal ablation methods, PFA minimizes the risk of damaging nearby structures, such as the esophagus, while maintaining high effectiveness. However, standardized methods for isolating the left atrial posterior wall remain lacking. Our study investigates the application of the FARAPULSE system to enhance PeAF treatment outcomes with PFA, providing a potentially safer and more efficient approach.

**HIGHLIGHTS**

- First-pass LAPWI success rate of 80% using the FARAPULSE system
- No major complications; arrhythmia recurrence in only 16.5%
- Fluoroscopy-guided PFA feasible even without 3D mapping

## Introduction and background

Catheter ablation for atrial fibrillation (AF) is a cornerstone in managing AF, particularly for preventing recurrences and improving long-term rhythm control. Pulmonary vein isolation (PVI) remains the primary ablation strategy in both paroxysmal and persistent AF (PeAF), achieved through the creation of linear lesions around the pulmonary vein (PV) antra. However, compared with paroxysmal AF, PeAF is more challenging to treat due to its greater atrial remodeling, longer arrhythmia duration, and a more complex arrhythmogenic substrate that extends beyond the PVs. These factors contribute to higher recurrence rates and lower success rates with PVI alone, underscoring the need for adjunctive strategies such as left atrial posterior wall isolation (LAPWI). In patients with PeAF, more extensive ablation strategies have often been advocated to enhance procedural success rates and reduce arrhythmia recurrence^[1–3]^. One such strategy is LAPWI in addition to PVI. The rationale for LAPWI is rooted in the understanding that the posterior wall of the left atrium (LA) plays a crucial role in maintaining PeAF due to its structural and electrophysiological properties^[2]^. This region, often referred to as the “fifth pulmonary vein,” shares embryological origins with the PVs and exhibits similar arrhythmogenic potential. Ablating this region may provide a substrate modification that could enhance the efficacy of AF ablation^[2,3]^.

Traditional LAPWI has been performed using thermal energy sources, such as point-by-point radiofrequency (RF) ablation or cryoballoon ablation. While these approaches have been extensively studied and widely utilized, their effectiveness in improving PeAF outcomes remains a matter of controversy. Some studies suggest that LAPWI, when performed in conjunction with PVI, can improve long-term freedom from AF, whereas others have reported no significant benefit compared to PVI alone^[4,5]^. These conflicting results may be attributed to variations in procedural techniques, patient selection, and differences in defining successful LAPWI. The absence of a standardized method for achieving durable electrical isolation of the left atrial posterior wall further complicates the interpretation of these findings^[6,7]^. Despite the potential benefits of LAPWI, the procedure is not without risks. A major concern associated with LAPWI is the risk of atrioesophageal fistula, a rare but life-threatening complication. Atrioesophageal fistula occurs due to thermal injury to the esophagus, which lies near the posterior wall of the LA. While the incidence of atrioesophageal fistula has been traditionally reported to be low, ranging from 0.02 to 0.1%, it carries a high mortality rate, estimated between 50 and 83%^[1,8]^. A recent European survey reported an incidence of 0.025% for atrioesophageal fistula in AF ablation procedures, with most cases associated with RF ablation (0.038%). The overall mortality rate in these cases was 65.8%^[9]^. These findings underscore the need for alternative ablation technologies to achieve effective lesion formation while minimizing collateral damage to surrounding structures.

Among these emerging technologies, pulsed-field ablation (PFA) has garnered increasing attention due to its unique nonthermal mechanism of action. PFA works through irreversible electroporation, in which high-voltage electrical pulses induce nanopore formation in cell membranes, leading to selective myocardial cell death without thermal injury. Unlike RF and cryoablation, which rely on thermal injury, PFA selectively ablates myocardial tissue while sparing adjacent noncardiac structures such as the esophagus, phrenic nerve, and PVs^[1,2,10]^. This selective tissue targeting makes PFA particularly appealing for LAPWI, where the risk of esophageal injury remains a significant concern. Several studies have demonstrated the efficacy and safety of PFA for PVI, showing comparable or superior acute success rates compared to conventional thermal ablation techniques. However, data on PFA for LAPWI in PeAF patients remains limited. The feasibility of extending PFA beyond PVI to include LAPWI is of particular interest, given its potential to enhance the success of AF ablation while reducing the risk of complications^[11,12]^. Initial preclinical and clinical studies suggest that PFA may achieve durable LAPWI without causing significant damage to the esophagus or surrounding tissues.

However, further research is needed to validate these findings and establish standardized protocols for PFA-based LAPWI^[13,14]^. In this context, the FARAPULSE PFA system (Boston Scientific) has emerged as a promising tool for catheter-based PFA. This system has been primarily studied for PVI but has shown potential for broader applications, including LAPWI. The unique design of the FARAPULSE catheter allows for efficient energy delivery and lesion formation while minimizing thermal spread. Investigating the role of PFA in LAPWI using this system could provide valuable insights into the safety, efficacy, and long-term outcomes of nonthermal posterior wall ablation in patients with PeAF^[15–17]^.

## Data collection

We conducted a thorough literature search to gather relevant studies, clinical trials, and review articles related to PFA for PVI and LAPWI in patients with PeAF. The search encompassed PubMed, Embase, Scopus, and Google Scholar from January 2000 to May 2023, using Boolean combinations of the following terms: (“pulsed field ablation” OR “irreversible electroporation”) AND (“atrial fibrillation” OR “AF”) AND (“pulmonary vein isolation” OR “PVI”) AND (“posterior wall isolation” OR “LAPWI”) AND (“recurrence” OR “outcome”). Additional manual searches were performed by reviewing the reference lists of relevant articles to identify missed studies.

Only peer-reviewed studies published in English were considered. Titles and abstracts were screened independently by two reviewers, with disagreements resolved by consensus. A total of 1140 records were initially retrieved, of which 96 full texts were assessed for eligibility, and 32 studies were ultimately included in the review. The inclusion criteria encompassed randomized controlled trials, cohort studies, and high-quality reviews reporting on PFA in PeAF patients, specifically assessing efficacy (e.g., freedom from arrhythmia) and safety outcomes (e.g., esophageal injury, stroke). Exclusion criteria included studies lacking full text, those in non-English languages, conference abstracts without supporting data, and those limited to animal or *ex vivo* models.

Data extraction was performed using a standardized template that captured study design, patient population, procedural techniques, ablation endpoints, follow-up duration, and reported outcomes. The primary outcomes of interest were procedural success (confirmed PVI and/or LAPWI) and long-term arrhythmia-free survival. Safety outcomes included periprocedural complications such as stroke, pericardial effusion, atrioesophageal fistula, and vascular injury.

Although this is a narrative review, we applied key elements of systematic methodology to enhance rigor. The methodological quality and risk of bias of the included studies were assessed qualitatively based on study design, sample size, and clarity of outcome reporting, consistent with AMSTAR-2 recommendations. Studies were grouped by procedural strategy (PVI-only vs. PVI + LAPWI), and comparisons were synthesized descriptively.

In particular, the ATHENA registry was included as a key large-scale, prospective, multicenter observational study designed to evaluate the real-world safety and effectiveness of pulsed field ablation. Patients with paroxysmal and PeAF were consecutively recruited across participating centers, with eligibility determined by standard clinical indications for ablation. Key inclusion criteria comprised adults with symptomatic AF referred for first-time or repeat catheter ablation, while exclusion criteria encompassed prior left atrial surgical interventions, contraindications to anticoagulation, and significant structural heart disease precluding ablation. Follow-up protocols involved scheduled clinic visits, ECG and Holter monitoring at 3, 6, and 12 months, and adverse event reporting. The registry methodology provided a robust framework to assess both acute procedural endpoints and long-term arrhythmia-free survival, complementing controlled trial data by capturing outcomes in a broader, unselected patient population. The ATHENA registry was conducted in accordance with the Declaration of Helsinki, with institutional review board approval at all participating centers and written informed consent obtained from all patients prior to enrollment.

**Figure 1.**
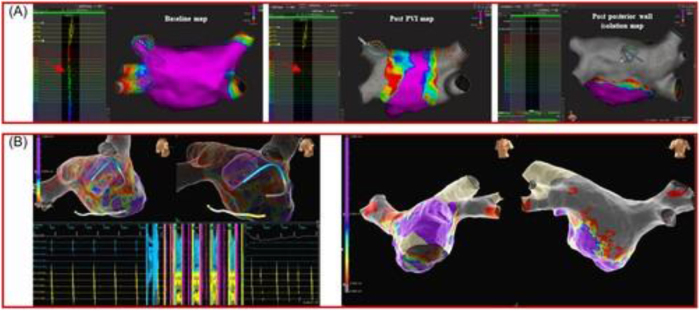
Validation of LAPW isolation using 3D mapping in *de novo* and redo cases, with Panel (A) showing Rhythmia mapping and Panel (B) NavX validation. Conduction gaps were identified during coronary sinus pacing, with further assessment through voltage and activation mapping.

**Figure 2.**
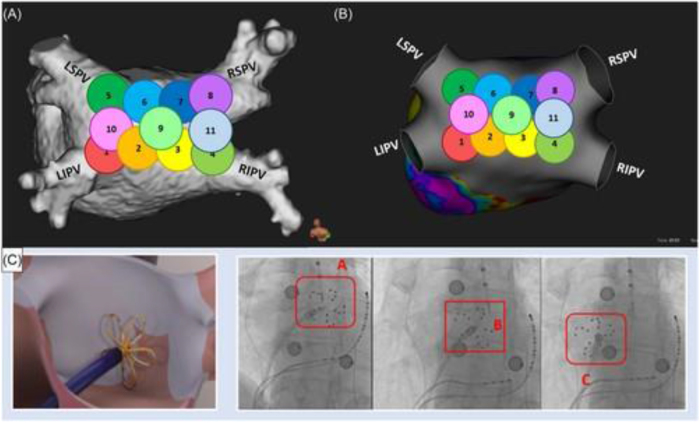
Following PVI, LAPW isolation was performed at the operator’s discretion using PFA to create continuous lesions linking the floor, roof, and inner wall of the LAPW, with additional ablation applied at carina sites when necessary. Panel (A) shows a CT reconstruction of the left atrium, Panel (B), a 3D-mapping reconstruction, and Panel (C), a fluoroscopic visualization of the LAPW ablation process.

**Figure 3.**
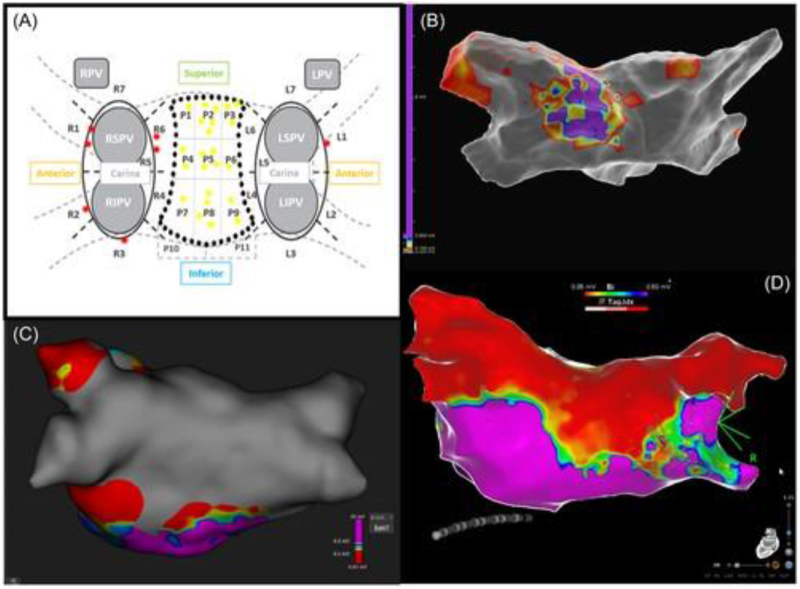
Lesion formation and residual gaps were analyzed by dividing PV encircling lines into seven sections and the LAPW into nine regions. Panel (A) highlights gap locations for PVs (red dots) and LAPW (yellow dots), Panel (B) shows residual atrial activity in the LAPW, and Panels (C) and (D) provide additional views of LAPW isolation results.

Additionally, technical details of the FARAPULSE PFA system were incorporated where available. The system utilizes a 12Fr over-the-wire balloon catheter, available in multiple balloon sizes (31 and 35 mm), with a flexible lattice-based electrode array designed for circumferential PV ablation. Energy delivery consists of a biphasic pulsed electric field waveform, typically delivered in microsecond-scale pulses grouped into trains, with pulse parameters calibrated to achieve irreversible electroporation of cardiomyocytes while sparing adjacent structures. Standard ablation protocols involve four applications per vein, delivered in a basket or flower configuration, with catheter positioning guided by fluoroscopy and electroanatomical mapping (EAM). The system is used in conjunction with a dedicated generator and sheath, allowing controlled energy delivery and catheter stability during lesion formation. Representative ablation workflows and lesion validation approaches are illustrated in Figures 1–3. Figure 1 demonstrates validation of LAPW isolation using 3D mapping systems, while Figure 2 depicts LAPW isolation strategies following PVI. Figure 3 highlights lesion formation patterns and gap analysis, with panels illustrating residual activity and region-specific ablation outcomes. Overly technical procedural descriptions (e.g., specific catheter deployment techniques, mapping systems, and lesion patterns) were excluded to maintain clarity and focus on clinically relevant endpoints. Descriptive statistics and reported outcome frequencies were used to summarize findings across studies, and figures were included to illustrate trends in ablation strategy and reported results.

### Statistical methods

Continuous variables were expressed as mean ± standard deviation or median with interquartile range, depending on distribution assessed by the Shapiro–Wilk test. Categorical variables were presented as counts and percentages. Comparisons between groups were performed using the Student’s *t*-test or Mann–Whitney *U* test for continuous variables and the *χ*^2^ test or Fisher’s exact test for categorical variables. Time-to-event outcomes (e.g., arrhythmia recurrence) were analyzed with Kaplan–Meier survival estimates and compared using the log-rank test. A two-sided *P*-value < 0.05 was considered statistically significant. Where available, *post hoc* power calculations were reported to confirm the adequacy of the sample size for detecting clinically meaningful differences. Statistical analyses were conducted using SPSS software (version 29.0, IBM Corp., Armonk, New York, USA).

Finally, in line with best practices for scientific integrity and the responsible integration of digital tools in research, we acknowledge the TITAN Guidelines 2025 for transparency in the reporting of artificial intelligence while noting that no generative AI tools were used in the preparation of our work^[18]^.

## Results

All statistical comparisons and *P*-values reported below were derived according to the tests specified in the Statistical methods section. The study population comprised 249 consecutive patients who underwent ablation procedures, with detailed clinical and procedural data presented in Tables 1 and 2. Among these patients, 54 individuals (21.7%) had a history of long-standing PeAF. The majority of the study participants were male (*n* = 198, 79.5%), with a mean age of 63 ± 9 years (see Table 1)^[1]^. The majority of procedures (*n* = 211, 84.7%) were *de novo* AF ablation, whereas 38 (15.3%) were repeat procedures. Before the index procedure, only 28.5% of the patients were in sinus rhythm. Approximately one-third of the procedures (*n* = 82, 32.9%) utilized a three-dimensional (3D) mapping system, and 75 (30.1%) were intracardiac echocardiography (ICE)-guided^[2]^.

**Table 1:** Baseline characteristics of the study population, including demographics, clinical history, and pre-procedural factors

Parameter	Overall population (*n* = 249)	Redo procedures (*n* = 38)	*De novo* PVI only (*n* = 93)	*De novo* PVI plus additional lesion set (*n* = 118)	*P* (*de novo* PVI only vs. *de novo* additional lesion set)
Age, years	63.4 ± 9	62.4 ± 7	63.4 ± 9	64.4 ± 8	0.1738
Female gender, *n* (%)	51 (20.5)	3 (7.9)	22 (23.7)	26 (22)	0.8689
Indication for ablation					
Persistent AF, *n* (%)	195 (78.3)	26 (68.4)	77 (82.8)	92 (78.0)	0.4876
Long-standing persistent AF, *n* (%)	54 (21.7)	12 (31.6)	16 (17.2)	26 (22.0)	
Symptomatic patient, *n* (%)	228 (91.6)	36 (94.7)	82 (88.2)	110 (93.2)	0.2317
History of AT/AFL					
	13 (5.2)	5 (13.2)	3 (3.2)	5 (4.2)	1
AT only, *n* (%)	3 (1.2)	1 (2.6)	1 (1.1)	1 (0.8)	
AFL only, *n* (%)	9 (3.6)	3 (7.9)	2 (2.2)	4 (3.4)	
Both AT and AFL, *n* (%)	1 (0.4)	1 (2.6)	0 (0.0)	0 (0.0)	
LVEF, %	53.7 ± 10	50.6 ± 11	55.2 ± 10	53.4 ± 9	0.2071
Left atrial volume, mL	42.6 ± 13	47.8 ± 13	42.3 ± 14	42.0 ± 13	0.92
Structural heart disease, *n* (%)	19 (7.6)	3 (7.9)	10 (10.8)	6 (5.1)	0.189
Coronary artery disease, *n* (%)	38 (15.3%)	5 (13.2)	14 (15.1)	19 (16.1)	0.8515
History of heart failure, *n* (%)	15 (6.0)	1 (2.6)	9 (9.7)	5 (4.2)	0.1631
CKD, *n* (%)	6 (2.4)	1 (2.6)	4 (4.3)	1 (0.8)	0.172
COPD, *n* (%)	14 (5.6)	0 (0.0)	3 (3.2)	11 (9.3)	0.0973
Hyperthyroidism, *n* (%)	12 (4.8)	3 (7.9)	0 (0.0)	9 (7.6)	0.0051
Sleep apnea, *n* (%)	10 (4.0)	1 (2.6)	4 (4.3)	5 (4.2)	1
Diabetes, *n* (%)	50 (20.1)	4 (10.5)	16 (17.2)	30 (25.4)	0.1802
Hypertension, *n* (%)	150 (60.1)	22 (57.9)	49 (52.7)	79 (66.9)	0.0467
History of major bleeding, *n* (%)	4 (1.6)	0 (0.0)	2 (2.2)	2 (1.7)	1
Antiarrhythmic, *n* (%)	158 (63.5)	20 (52.6)	67 (72.0)	71 (60.2)	0.0813
Beta-blockers, *n* (%)	168 (67.5)	31 (81.6)	65 (69.9)	72 (61.0)	0.1937

**Table 2:** Procedural parameters of the study population, including ablation modality (PFA or conventional), use of imaging and mapping guidance, anesthesia type, procedure duration, and fluoroscopy time

Parameter	Overall population (*n* = 249)	Redo procedures (*n *= 38)	*De novo* PVI only (*n* = 93)	*De novo* PVI plus additional lesion set (*n* = 118)	*P* (*de novo* PVI only vs. *de novo* additional lesion set)
Ablation approach					
*De novo, n* (%)	211 (84.7)	0 (0.0)	93 (100)	118 (100)	1
Repeated ablation, *n* (%)	38 (15.3)	38 (100)	0 (0.0)	0 (0.0)	
Anesthesia protocol					
GA	140 (56.2)	18 (47.4)	43 (46.2)	79 (66.9)	0.003
Deep sedation	109 (43.8)	20 (52.6)	50 (53.8)	39 (33.1)	
Ablation target					
PVI only, *n* (%)	106 (42.6)	13 (34.2)	93 (100)	0 (0.0)	<0.0001
PVI plus additional lesions, *n* (%)	142 (57.0)	24 (62.2)	0 (0.0)	118 (100)	
Additional lesions only, *n* (%)	1 (0.4)	1 (2.6)	0 (0.0)	0 (0.0)	
Mapping system used	82 (32.9)	16 (42.1)	12 (12.9)	54 (45.8)	<0.0001
Carto, *n* (%)	20 (24.4)	2 (12.5)	1 (8.3)	17 (31.5)	
Ensite NavX, *n* (%)	23 (28.0)	8 (50.0)	4 (33.3)	11 (20.4)	
Rhythmia, *n* (%)	39 (47.6)	6 (37.5)	7 (58.3)	26 (48.1)	
Intracardiac echocardiography, *n* (%)	75 (30.1)	14 (36.8)	17 (18.3)	44 (37.3)	0.0031
Sinus rhythm at the procedure, *n* (%)	71 (28.5)	11 (28.9)	43 (46.2)	17 (14.4)	<0.0001
Cardioversion attempt	127 (51.0)	20 (52.6)	43 (46.2)	64 (54.2)	0.1738
Successful, *n* (%)	113 (89.0)	16 (80.0)	39 (90.7)	58 (90.6)	1
Unsuccessful, *n* (%)	14 (11.0)	4 (20.0)	4 (9.3)	6 (9.4)	
Sinus rhythm at the end of the procedure, *n* (%)	230 (92.4)	38 (100)	92 (98.9)	100 (84.7)	0.0002
LA anatomy variants	13 (5.2)	5 (13.2)	6 (6.5)	2 (1.7)	0.1422
Left common trunk, *n* (%)	6 (2.4)	3 (7.9)	2 (2.2)	1 (0.8)	
Right middle pulmonary vein, *n* (%)	7 (2.8)	2 (5.3)	4 (4.3)	1 (0.8)	
Lab occupancy time, min	110 [80–145]	145 [100–174]	100 [70–133]	110 [80–140]	0.0934
Support time (preparation plus skin-to-skin), min	85 [70–120]	95 [71–150]	78 [65–118]	90 [70–113]	0.0828
Skin-to-skin (primary operator) time, min	70 [60–90]	80 [57–104]	68 [60–90]	70 [59–88]	0.6205
Total fluoroscopy time, min	17 [13–23]	14 [8–24]	17 [13–24]	17 [14–22]	0.7412
LA dwell time, min	18 [14–25]	20 [16–27]	15 [12–20]	22 [17–28]	<0.0001
Number of PFA deliveries at PVs, *n*	32 [32–38]	32 [32–46]	32 [32–40]	32 [32–34]	<0.0001
Number of PFA deliveries outside of PVs, *n*	16 [14–21]	16 [12–21]	0 [0–0]	16 [14–21]	<0.0001
Number of PFA deliveries, *n*	46 [32–52]	49 [38–60]	32 [32–40]	50 [46–56]	0.0552

The decision to perform PVI alone or PVI with additional LAPWI was influenced by patient-specific clinical characteristics. The PVI-only group had more patients requiring electrical cardioversion at least once (*n* = 40, 47.6%) compared to the PVI + LAPWI group (*n* = 68, 60.4%, *P* = 0.035). Additionally, the PVI-only group had a higher rate of antiarrhythmic drug (AAD) failure at baseline (*n* = 67, 72%) compared to the PVI + LAPWI group (*n* = 71, 60.2%, *P* = 0.049). In the LAPWI group, 54 cases (45.8%) were approached with a 3D mapping system, while 44 (37.3%) utilized ICE. The employment of 3D mapping showed a decline over time, as evidenced by a reduction in utilization across four consecutive quarters (*n* = 21 in the first quarter, *n* = 16 in the second quarter, *n* = 10 in the third quarter, and *n* = 7 in the fourth quarter). The majority of ablation procedures were conducted under general anesthesia (*n* = 140, 56.2%), while deep sedation was used in the remaining 109 cases (43.8%)^[3]^. The baseline characteristics of patients undergoing *de novo* PVI-only versus *de novo* PVI with additional lesion sets were comparable, as presented in Table 2.

PVI was achieved in all patients (100%) using PFA exclusively, with a median of 32 PFA applications per patient^[4]^. Anatomical variations in the LA were noted in 5.2% of cases, with seven cases (2.8%) of right middle PV and six cases (2.4%) of left common trunk PV. First-pass isolation was successful in 98.0% of veins, as confirmed by entrance and exit block assessments via 3D mapping. Seven residual PV conduction gaps were identified, including four at the right superior PV, two at the right inferior PV, and one at the left superior PV, as shown in Figure 3A. Additional PFA applications were required in 57.6% (*n* = 143) of cases, targeting areas outside the PVs, particularly the LAPW. These procedures necessitated a median of 16 [14–20.5] PFA applications, achieving complete LAPWI in all cases, validated through differential pacing and/or 3D mapping^[5]^.

Among patients undergoing *de novo* PVI + LAPWI, 50 (42.4%) underwent complete 3D mapping to verify PVI and LAPWI success. The mean ablated left atrial posterior wall area was 20.0 ± 6 cm^2^. First-pass block of the roof area (sectors #1–#3) was achieved in 44 patients (88%), while the floor area (sectors #7–#9) exhibited first-pass block in 43 cases (86%), and the inner area (sectors #3–#6) showed efferent block in 45 cases (90%). Overall, first-pass LAPWI was achieved in 40 patients (80%). A total of 23 residual atrial conduction gaps were identified through high-output pacing and 3D mapping validation. The PFA splines demonstrated an accuracy of 94.9% (437 of 450 sectors) in detecting atrial conduction when compared with subsequent high-output pacing assessments. Pace-mapping post-PFA was confirmatory with voltage and propagation mapping in all cases (100%) (see Fig. 3A). Examples of residual left atrial posterior wall conduction and subsequent successful PFA consolidation are depicted in (Fig. 3B–D). Following additional PFA applications, LAPWI was achieved in all cases (100%), validated by differential pacing and/or 3D mapping^[6]^.

Skin-to-skin procedural duration was comparable between PVI-only and PVI + LAPWI groups (median 68 [60–90] vs. 70 [58–88] minutes; *P* = 0.620). Lab occupancy time also showed no significant difference (100 [70–133] vs. 110 [80–140] minutes; *P* = 0.093). Fluoroscopy time was similar between the groups (17 [14–22] vs. 17 [13–24] minutes; *P* = 0.741), as was the number of PFA deliveries required for PVI (32 [32–34] vs. 32 [32–40] applications; *P* = 0.055)^[7]^.

**Figure 4.**
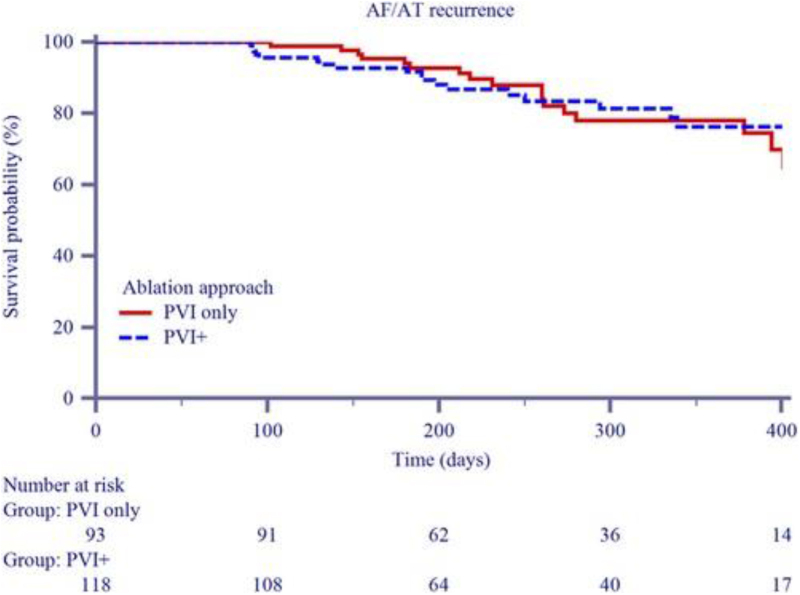
Kaplan–Meier curves illustrating arrhythmia recurrence rates during follow-up, stratified by ablation strategy, with red indicating PVI-only and blue PVI + LAPWI.

No major periprocedural complications, including stroke, transient ischemic attack, or cardiac tamponade, were reported. Additionally, there were no anesthesia-related complications. Across the entire population of 249 patients, 6 individuals (2.4%) experienced minor complications, all of which were groin hematomas. Of these, three occurred in the PVI-only group (*n* = 133, 2.3%) and three in the PVI + LAPWI group (*n* = 116, 2.6%), with no statistically significant difference between groups. Conservative management and medical therapy were sufficient in all cases, with no extended hospital stays required. During a median follow-up of 273 [191–379] days, 41 patients (16.5%) experienced a recurrence of AF, atrial tachycardia (AT), or atrial flutter (AFL) beyond the 90-day blanking period. Kaplan–Meier survival analysis (Fig. 4) demonstrated freedom from arrhythmia recurrence at 6 months of 85.1% in the PVI-only group and 86.7% in the PVI + LAPWI group (log-rank *P* = 0.58). Median time to recurrence was 223 ± 100 days, and no significant differences were observed between ablation strategies. Stratification by AF type revealed recurrence rates of 15.9% for PeAF and 18.5% for long-standing PeAF (*P* = 0.682). In the redo group, five recurrences (13.2%) were observed, with no significant difference compared to either the PVI-only (*P* = 0.610) or PVI + LAPWI groups (*P* = 1.000)^[8]^.

## Discussion

### Main findings

Our study provides a detailed account of the ablation workflow used for LAPWI with the PFA system, utilizing either fluoroscopic guidance alone or validation through a high-density mapping system. It represents one of the most extensive analyses of PeAF patients undergoing LAPWI with this technique. Our main findings highlight the feasibility of achieving LAPWI with the FARAPULSE PFA catheter, as electrical isolation was obtained in all cases. A first-pass isolation rate of 80% was observed, eliminating the need for additional PFA or RF touch-ups following remapping and signal assessments with pacing maneuvers^[1-3]^.

When performed alongside PVI, LAPWI using this PFA catheter did not lead to a significant prolongation of procedural duration compared to PVI-only cases. The procedure’s safety profile remained favorable, with no major complications observed during or after the procedure. Minor complications occurred at a low rate of 2.4%, with no significant differences noted across patient groups^[1,4,5]^. During a median follow-up period of 273 days, arrhythmic recurrences were reported in 16.5% of patients. No significant differences emerged when analyzing outcomes based on AF pattern or ablation strategy. These included comparisons among persistent and long-standing PeAF patients and those who underwent PVI-only, PVI combined with LAPWI, or repeat ablation procedures^[6]^.

### PFA energy for PW isolation

A growing body of evidence suggests that PFA technology can be effectively utilized for LAPWI in patients with PeAF, though only a limited number of cases have been documented so far. Early feasibility studies demonstrated that the pentaspline PFA catheter, when maneuvered in a flower configuration, allowed for effective positioning along the posterior LA^[1,7,8]^. Its shape and flexibility enabled operators to achieve comprehensive PW coverage with minimal repositioning. While EAM has been routinely employed alongside fluoroscopic and ICE guidance to visualize catheter placement, our findings indicate that LAPWI can be achieved even with fluoroscopic guidance alone. This represents a significant step forward, as we observed a decline in the use of 3D mapping systems over time, with operators increasingly relying on fluoroscopic imaging to ensure adequate lesion delivery^[9-11]^.

By capturing static fluoroscopic images of catheter positioning before advancing to another area, operators could achieve consistent LAPWI without necessitating real-time mapping integration. This approach provided confidence in the ablation workflow, reducing the perceived need for 3D mapping validation in later study phases. However, when mapping was utilized, electroanatomical visualization of the catheter splines facilitated the monitoring of PFA energy delivery, ensuring optimal lesion formation and effective PW coverage^[12–14]^. The mapping-validated cohort allowed for a more precise assessment of left atrial posterior wall lesion continuity, confirming the effective connection between the floor, roof, and inner left atrial posterior wall regions. Conduction gaps were most frequently detected in central left atrial posterior wall areas, particularly in regions far from prior PV energy deliveries. These gaps, identified in more than half of the cases within specific left atrial posterior wall zones, were successfully addressed using additional PFA applications, eliminating the need for RF touch-ups. Even in cases of atypical PV anatomy, such as the presence of a left common trunk or a right accessory PV, the PFA system alone was sufficient to achieve complete LAPWI, demonstrating its adaptability and efficacy in complex anatomical presentations^[15–17]^.

### Clinical implications and safety concerns

PVI using PFA has demonstrated feasibility and safety, with a recent randomized trial confirming its noninferiority to thermal ablation. However, a PVI-only approach may not always provide optimal arrhythmia control, particularly in PeAF. Studies have reported higher recurrence rates in these patients when balloon-based PVI is employed^[19,20]^. The left atrial posterior wall shares an embryological origin with the PVs, forming a contiguous structure in the LA. This has led to speculation about whether LAPWI should be incorporated into PeAF ablation strategies. A recent randomized trial suggested that adding RF LAPWI to PVI may not offer additional benefits unless rapid left atrial posterior wall electrical activity is detected during EAM. However, these findings remain inconclusive, as previous attempts to identify key focal sources of PeAF, such as rotors and focal drivers, have not consistently yielded a definitive mapping approach. Some trials have shown that LAPWI can improve long-term procedural outcomes over PVI alone, highlighting the need for further investigation^[21–23]^.

In the CAPLA trial, all patients underwent point-by-point box LAPWI with RF after PVI, with mapping and pacing used to confirm acute LAPWI. The box lesion set, while widely used, has been criticized for its susceptibility to electrical gaps, which may lead to reconnection of the left atrial posterior wall over time if lesions are inconsistent^[1,24]^. Additionally, this lesion set may not sufficiently isolate arrhythmogenic sources that extend beyond the defined ablation zone, potentially leading to higher rates of post-ablation atrial arrhythmias. The LIBERATION trial demonstrated that complete LAPWI in combination with PVI was superior to PVI alone, likely due to reducing critical left atrial mass and minimizing reconnections. However, this strategy may prolong procedural duration and increase the risk of periprocedural complications due to the higher energy deliveries required at the left atrial posterior wall level. The CAPLA trial reported procedural times of 142 min for achieving LAPWI using a box lesion set, whereas LAPWI procedures in our study averaged 90 min^[1,25,26]^.

PFA technology has the potential to address some of these challenges. By achieving uniform and complete LAPWI, PFA may mitigate the issue of electrical gaps associated with traditional point-by-point ablation techniques. Such gaps have been a major contributor to left atrial posterior wall reconnection and suboptimal outcomes. Additionally, PFA may offer a safer alternative to RF ablation by reducing concerns about esophageal injury, a significant risk when extensive RF ablation is performed at the left atrial posterior wall^[27,28]^.

The esophagus lies in close anatomical proximity to the posterior left atrial wall, making it particularly vulnerable during thermal ablation procedures. RF energy can result in collateral heat transfer, increasing the risk of esophageal ulceration, thermal injury, or, in rare cases, atrioesophageal fistula – a life-threatening complication. In contrast, PFA’s nonthermal mechanism of action relies on electroporation, which preferentially affects myocardial tissue while sparing adjacent structures, including the esophagus.

Several preclinical and early clinical studies have demonstrated that PFA significantly reduces or eliminates esophageal thermal damage, with some trials reporting no detectable esophageal injury post-procedure^[28]^. Despite these findings, further long-term and high-resolution imaging studies are warranted to fully confirm the esophageal safety profile of PFA in broader patient populations.

Another advantage of PFA lies in its ability to penetrate adipose tissue, which is particularly relevant along the left atrial roof. The subepicardial septo-pulmonary bundle in this region is often separated from the subendocardial septo-atrial bundle by a variable layer of adipose tissue, which has low electrical conductivity and may insulate deeper structures from conventional RF ablation. The limited thermal conductivity of adipose tissue further restricts lesion formation, making transmural ablation challenging. Our study did not detect significant differences between PVI-only and PVI + LAPWI groups, aligning with findings from recent studies. However, the relatively small sample size and short follow-up duration may have limited our ability to detect long-term differences in treatment efficacy^[1,29,30]^.

The potential benefits of LAPWI may become more apparent over an extended follow-up period. Aryana *et al* reported no significant difference in arrhythmia recurrence at 12 months between PVI-only and PVI + LAPWI groups^[7]^. However, at 39 months, patients who underwent combined PVI + LAPWI had significantly higher rates of freedom from all atrial arrhythmias and AF compared to those who received PVI alone. This suggests that while short-term outcomes may appear similar, the additional effect of LAPWI on reducing long-term arrhythmia recurrence should not be overlooked^[1,31,32]^.

When comparing our results to those of the PIVOTAL AF trial, we observed a lower recurrence rate among patients with PeAF treated with PVI-only in our cohort. The difference may be attributed to variations in follow-up duration and monitoring strategies. The PIVOTAL trial employed transtelephonic monitoring, which is likely more sensitive in detecting AF recurrence than the methodology used in our study^[33–35]^. In contrast, our study found recurrence rates among PVI + LAPWI patients to be comparable to those reported by Badertscher *et al*, who observed a 15% recurrence rate over a median follow-up of 144 days. This reinforces the notion that LAPWI may enhance the ablation efficacy of PVI alone. However, it is essential to recognize that procedural techniques can impact outcomes. In the study by Badertscher *et al*, first-pass isolation was confirmed using a multipolar mapping catheter. In contrast, our approach relied solely on fluoroscopy in more than half of the cases^[1,36,37]^.

Preclinical data further support the advantages of PFA over RF ablation. Histopathological studies have shown that PFA lesions consist of organized, homogeneous fibrosis with well-demarcated borders, while RF-induced lesions tend to be more heterogeneous, with a greater inflammatory response^[1,2,38]^. This distinction may have implications for post-ablation outcomes. High-density mapping studies have demonstrated that PFA lesions are uniform, with minimal fractionation at lesion borders, which may reduce the proarrhythmic effects associated with incomplete or suboptimal left atrial posterior wall ablation using RF energy. The absence of fragmented electrical activity at lesion margins may help prevent the development of post-ablation ATs, further underscoring the potential of PFA as a superior strategy for LAPWI^[39–41]^.

### Synthesis of literature

AF is the most prevalent sustained arrhythmia, associated with increased risks of stroke, heart failure, and mortality^[1,2]^. AF subtypes include paroxysmal, persistent, and long-standing persistent forms, with PeAF posing greater therapeutic challenges due to progressive atrial remodeling^[3]^. Management focuses on stroke prevention, symptom control, and maintaining a stable rhythm. While rate control and anticoagulation, guided by the CHA_2_DS_2_-VASc score (see Table 3), remain essential, rhythm control with AADs is limited by modest efficacy and adverse effects^[4,5]^.

**Table 3:** Structured comparison of different atrial fibrillation management strategies, including symptom control, stroke prevention, and rhythm maintenance

Therapeutic strategy	Mechanism of action	Symptom control	Stroke prevention	Maintenance of sinus rhythm	Examples	Advantages	Limitations	Clinical considerations
Beta-blockers	Reduces heart rate by blocking β-adrenergic receptors	✓	✗	✗	Metoprolol, Atenolol	Effective for rate control	Can cause fatigue and hypotension	Preferred in patients with hypertension or heart failure
Calcium channel blockers	Slows AV node conduction	✓	✗	✗	Diltiazem, Verapamil	Useful in patients intolerant to beta-blockers	Avoid in heart failure with reduced EF	Best for younger patients with normal LV function
Anticoagulation therapy	Prevents thrombus formation	✗	✓	✗	Warfarin, DOACs (Apixaban, Rivaroxaban)	Reduces stroke risk	Increased bleeding risk	Based on CHA_2_DS_2_-VASc score
Antiarrhythmic drugs (AADs)	Modulates cardiac ion channels	✗	✗	✓	Amiodarone, Flecainide, Sotalol	Effective in maintaining sinus rhythm	Proarrhythmic effects, toxicity	Monitor QT interval, liver and thyroid function
Catheter ablation	Destroys arrhythmogenic foci via RF or PFA	✓	✗	✓	Radiofrequency (RF), Pulsed Field Ablation (PFA)	Superior to AADs for rhythm control	Requires specialized centers	Ideal for symptomatic AF resistant to AADs
AV node ablation and pacing	Eliminates AV conduction and paces ventricles	✓	✗	✗	Pacemaker implantation	Useful in refractory AF	Permanent pacemaker dependency	Reserved for patients unresponsive to other therapies
Left atrial appendage occlusion (LAAO)	Blocks clot formation in LAA	✗	✓	✗	Watchman Device	Alternative to anticoagulation	Requires procedural expertise	Recommended in high-bleeding-risk patients
Lifestyle modifications	Targets underlying risk factors	✓	✓	✓	Weight loss, alcohol reduction	Improves overall outcomes	Requires long-term adherence	Essential for all AF patients
Hybrid approaches	Combines multiple treatment modalities	✓	✓	✓	AADs + Ablation, LAAO + Anticoagulation	Maximizes success rates	May involve multiple procedures	Personalized based on patient profile

PVI has emerged as the cornerstone of catheter ablation, targeting ectopic triggers within myocardial sleeves of the PVs^[1,2]^. However, in PeAF, structural and electrical remodeling – including fibrosis, altered ion channel function, and autonomic imbalance – extend the arrhythmic substrate beyond the PVs, often necessitating adjunctive ablation^[3-5]^.

The left atrial posterior wall has been identified as a key contributor to AF maintenance due to shared embryological origins with the PVs, high arrhythmogenic potential, and observed conduction abnormalities^[1-3]^. Electroanatomical studies reveal high-frequency signals and slow conduction zones in this region. Multiple trials, including the CONVERGE trial, support the efficacy of combining PVI with LAPWI for improved rhythm outcomes in PeAF^[4]^.

However, traditional thermal techniques (RF and cryoablation) used for LAPWI carry risks, notably esophageal injury, due to the anatomical proximity of the esophagus to the posterior LA. These risks include atrioesophageal fistula, a rare but fatal complication^[5]^.

PFA offers a promising nonthermal alternative by inducing electroporation-mediated cardiomyocyte death while sparing adjacent tissues^[6,7]^. This selectivity reduces complications such as phrenic nerve injury and esophageal damage, as confirmed in preclinical and clinical studies^[8]^. Additionally, PFA avoids steam pops and minimizes tissue inflammation, enhancing procedural safety and potentially reducing atrial tachyarrhythmias^[9]^.

Durability of LAPWI remains a challenge, with traditional methods often leading to incomplete isolation or electrical reconnection. Novel single-shot PFA catheters, balloon technologies, and high-resolution mapping systems have been developed to improve lesion durability^[2,6]^. Hybrid strategies, such as the convergent approach combining endocardial and epicardial ablation, show promise in achieving more durable posterior wall isolation^[7]^.

Outcomes from randomized trials and registries indicate that adjunctive LAPWI improves freedom from AF, especially in persistent and long-standing persistent cases^[8]^. A recent meta-analysis demonstrated superior 1-year sinus rhythm maintenance when LAPWI was added to PVI^[9]^. Nevertheless, variability in outcomes persists due to differences in ablation technique, patient selection, and follow-up protocols. Concerns about proarrhythmic effects following incomplete LAPWI remain, warranting further investigation^[10]^. PFA’s unique advantages over thermal ablation include: (1) Tissue selectivity: Cardiomyocytes are more susceptible to electroporation than surrounding structures like nerves or the esophagus^[5]^. (2) Efficiency: Procedures are shorter due to rapid lesion formation and minimal need for repositioning. (3) Safety: Clinical trials such as IMPULSE, PEFCAT, and ADVENT report high PVI success (>98%) with significantly fewer complications compared to RF and cryoablation^[5-7]^.

**Figure 5.**
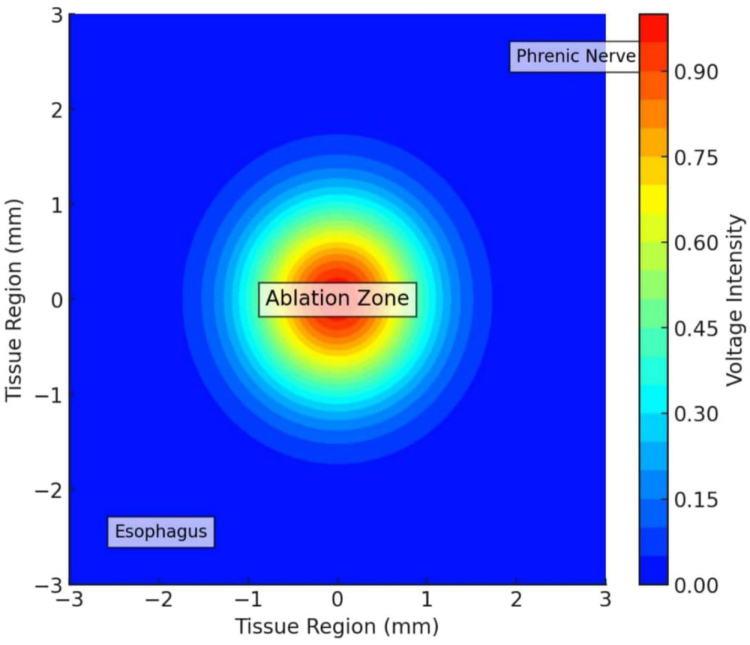
Electroporation selectivity illustrated by concentration of the electrical field within the ablation zone, with sparing of adjacent structures such as the esophagus and phrenic nerve.

**Figure 6.**
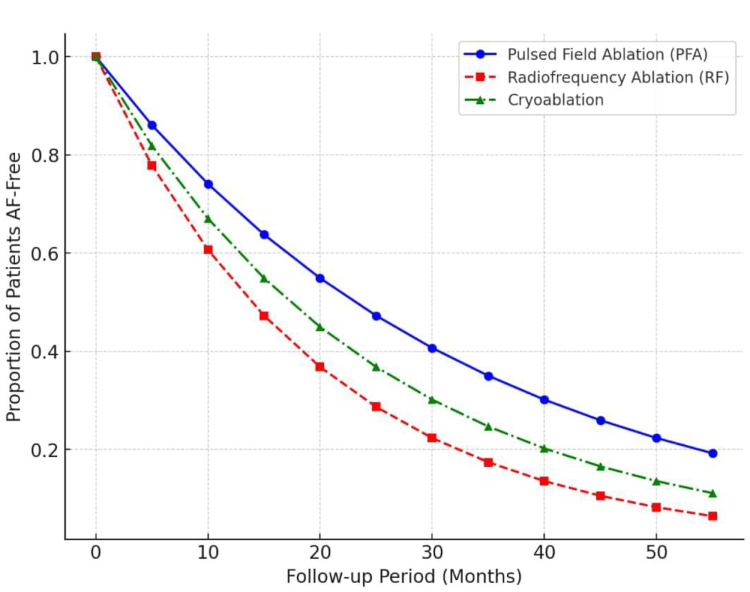
Kaplan–Meier curve illustrating AF-free survival following ablation. The figure demonstrates how PFA performs over time compared with other ablation methods.

Follow-up data show promising durability of PFA-based PVI and LAPWI. Acute success – defined by bidirectional PV isolation – has been nearly universal, with reduced collateral injury (see Fig. 5) ^[8]^. Freedom from AF at 6–12 months was 80% in paroxysmal AF and 65–70% in PeAF, aligning with or surpassing outcomes from thermal ablation (see Fig. 6)^[8,9]^. Additionally, PFA has been associated with fewer late PV reconnections and post-ablation atrial arrhythmias due to its noninflammatory mechanism.

Ongoing large-scale studies, including the MANIFEST-PF registry, will provide further insights into long-term efficacy, patient selection, and best practices for integrating PFA into ablation strategies^[10,11]^. However, while long-term data on PVI durability with PFA continue to grow, less is known about the sustainability of electrical isolation when this modality is applied to the left atrial posterior wall.

In this context, the ATHENA registry contributes unique insights by specifically evaluating PeAF patients undergoing LAPWI with the FARAPULSE system across multiple Italian centers^[3,10]^. Unlike MANIFEST-PF, which primarily focused on large-scale real-world PVI outcomes, or PIVOTAL, which concentrated on establishing initial safety and efficacy, ATHENA highlights the feasibility and outcomes of adjunctive LAPWI in a real-world PeAF population. This distinction underscores its role in expanding the evidence base for PFA beyond standard PVI, particularly in more challenging patient subsets^[10,11]^.

PFA has demonstrated high acute success rates in achieving LAPWI; however, evidence regarding the long-term durability of these lesions remains limited. Early clinical studies have shown promising short- to mid-term outcomes, with durable posterior wall isolation observed in some patients during remapping procedures conducted within 3–6 months post-ablation. However, beyond this timeframe, data are sparse, and there is considerable variability in lesion durability due to factors such as lesion depth, tissue characteristics, and atrial remodeling. Given the dynamic remodeling of atrial tissue and the potential for conduction recovery, especially in non-scarred myocardium, further studies are essential to confirm the lasting efficacy of LAPWI lesions created with PFA. Unlike thermal energy modalities, PFA creates nonthermal lesions through electroporation, which may result in different healing and reconnection patterns over time. This underscores the need for prospective, long-term follow-up studies using systematic remapping protocols to evaluate the persistence of electrical isolation and its correlation with arrhythmia-free survival. Until such evidence is established, conclusions about the long-term efficacy of PFA-based LAPWI should be interpreted with caution.

### Technological advances in pulsed field ablation

The evolution of PFA catheter designs has played a crucial role in its clinical adoption. Initially, monopolar PFA catheters were developed, generating unidirectional electrical fields that required precise electrode placement to achieve effective ablation^[41]^. However, bipolar catheters have since gained prominence due to their ability to create more uniform and controlled lesion sets. Bipolar PFA catheters deliver electric pulses between adjacent electrodes, reducing the risk of nonuniform ablation and improving lesion durability^[42]^. This advancement has particularly benefited PVI, where consistent lesion formation is critical for preventing AF recurrence.

Additionally, the development of focal PFA catheters has expanded the technology’s versatility, allowing for targeted ablation of specific arrhythmogenic substrates while minimizing unintended myocardial damage^[43]^. Another significant innovation in PFA catheter design is the introduction of balloon-based systems. Unlike focal catheters, which require multiple applications to achieve complete PVI, balloon catheters enable single-shot ablation, reducing procedural time and improving efficiency^[7]^. These balloon-based PFA systems incorporate multi-electrode arrays that distribute electric fields evenly, ensuring homogeneous tissue ablation. Moreover, some balloon catheters integrate real-time impedance monitoring to assess tissue contact and optimize energy delivery, further enhancing procedural success rates^[44]^. Advancements in mapping and navigation systems have also significantly improved PFA outcomes. Traditional EAM systems, which have been widely used for RF and cryoablation, have been adapted for PFA to provide high-resolution, real-time visualization of cardiac anatomy and electrical activity^[45]^. High-density mapping catheters allow precise identification of arrhythmogenic regions, ensuring targeted ablation with minimal unnecessary tissue damage.

Additionally, novel contact force-sensing catheters have been integrated into PFA procedures, providing real-time feedback on electrode-tissue interaction to optimize lesion formation and reduce complications^[46]^. The integration of AI and machine learning into PFA procedures represents a transformative step in the field of electrophysiology. AI-driven algorithms are being developed to assist in real-time mapping, automating the identification of ablation targets based on patient-specific arrhythmia patterns^[47]^. These algorithms analyze vast datasets from prior procedures to predict optimal ablation sites, improving procedural efficiency and standardizing outcomes across operators with varying experience levels. Moreover, AI-powered image processing techniques enhance pre-procedural planning by refining cardiac imaging modalities such as magnetic resonance imaging (MRI) and CT scans, facilitating better anatomical assessment before PFA procedures^[48]^. In addition to procedural guidance, machine learning models are used to predict post-ablation outcomes. These models can predict the likelihood of AF recurrence by analyzing patient demographics, comorbidities, and procedural parameters, enabling personalized patient management strategies^[49]^. AI-driven remote monitoring systems are also being explored, utilizing wearable technology and implantable loop recorders (ILRs) to track atrial arrhythmias following PFA continuously. These systems can facilitate early detection of AF recurrence, enabling timely interventions and ultimately improving long-term patient outcomes^[50]^.

### Patient selection and predictors of success

Ideal candidates for PFA-based PVI and LAPWI include patients with symptomatic PeAF who have failed or are intolerant to at least one AAD. The role of PFA in this population is advantageous due to its ability to create nonthermal lesions, thereby reducing the risk of PV stenosis, esophageal injury, and phrenic nerve palsy, which are common concerns associated with RF and cryoablation^[3]^. Patients with a structurally normal LA and without significant atrial fibrosis appear to derive the greatest benefit, as substrate modification in fibrotic atria can be less effective and may necessitate additional adjunctive strategies^[4]^. A key determinant of successful ablation outcomes is the burden and chronicity of AF. Patients with shorter AF durations, particularly those with paroxysmal or early stage PeAF, exhibit higher rates of sinus rhythm maintenance following ablation^[5]^. Conversely, long-standing PeAF, often defined as AF lasting more than 1 year, is associated with more extensive electrical and structural remodeling, which reduces the likelihood of procedural success and increases the probability of AF recurrence^[6]^. In such cases, additional substrate modification strategies, such as posterior wall isolation and adjunctive linear ablation, may be required to improve rhythm outcomes^[7]^.

Biomarkers and imaging modalities play a crucial role in patient selection and predicting the success of ablation. Elevated levels of natriuretic peptides (BNP, NT-proBNP) and inflammatory markers such as C-reactive protein have been associated with increased atrial remodeling and worse ablation outcomes, suggesting their utility in risk stratification^[8]^. Similarly, cardiac imaging techniques, including late gadolinium enhancement MRI, can quantify atrial fibrosis, a strong predictor of recurrent AF post-ablation^[9]^. The Utah classification, based on the extent of fibrosis detected on MRI, provides a valuable framework for patient selection, with minimal fibrosis (Utah stage 1) correlating with superior ablation success rates compared to advanced fibrotic stages (Utah stage 4)^[10]^. Echocardiographic parameters, such as left atrial volume index (LAVI) and left atrial strain, have also emerged as important predictors of ablation success. Increased LAVI is associated with greater atrial remodeling and a higher likelihood of AF recurrence, whereas preserved left atrial strain reflects better atrial function and improved procedural outcomes^[11]^. Additionally, transesophageal echocardiography is often performed pre-procedurally to assess for left atrial appendage thrombus, ensuring the safety of the ablation procedure^[12]^.

Electrophysiological characteristics further refine patient selection and predict the likelihood of procedural success. High-frequency fractionated electrograms and complex fractionated atrial electrograms (CFAEs) have been implicated in the maintenance of AF and are often targeted during substrate modification strategies^[13]^. Stable rotational activities, identified through electrogram mapping, have been linked to successful PVI and posterior wall isolation outcomes, particularly when using PFA^[14]^. However, the role of CFAE-guided ablation remains controversial, as recent studies suggest variable success rates compared to anatomical and voltage-guided approaches^[15]^. For patients with PeAF and significant atrial enlargement, adjunctive ablation strategies beyond PVI may be necessary to achieve rhythm control. LAPWI has been proposed as an effective adjunct, particularly in patients with evidence of posterior wall involvement in AF propagation^[16]^.

Studies have demonstrated that durable LAPWI, when combined with PVI, enhances freedom from AF compared to PVI alone, particularly in patients with PeAF and extensive atrial remodeling^[17]^. PFA has shown promise in achieving durable posterior wall isolation without the risk of thermal injury to adjacent structures, making it a preferred modality in this patient subset^[10]^. The consideration of redo procedures is essential to PFA-based ablation strategies, as recurrence rates in PeAF remain substantial despite initial procedural success. The mechanisms underlying AF recurrence include incomplete lesion formation, PV reconnection, and non-PV triggers^[19]^. Repeat procedures often target areas of conduction gaps and residual arrhythmogenic substrate, with PFA offering a potential advantage in achieving more durable lesion sets than conventional techniques^[20]^.

Recent studies suggest that most recurrences occur within the first 6 months post-ablation, highlighting the importance of close follow-up and early identification of recurrence patterns. ILRs and extended Holter monitoring have facilitated early detection of asymptomatic recurrences, allowing timely intervention when necessary^[21]^. Additionally, adjunctive pharmacological strategies, including short-term AAD therapy, have been explored to reduce early recurrences and improve long-term procedural success^[22]^. Assessing lesion durability and identifying residual conduction gaps is critical in patients undergoing redo ablation. High-resolution electroanatomic mapping systems provide detailed insights into conduction patterns and can guide targeted ablation of recurrent arrhythmogenic sites^[23]^. The role of PFA in redo procedures is under investigation, with preliminary data suggesting a favorable safety profile and efficacy in addressing PV reconnections and posterior wall breakthroughs^[24]^.

### Cost-effectiveness and healthcare utilization

A key driver of cost-effectiveness in PFA is its ability to minimize collateral damage to surrounding tissues, including the esophagus, phrenic nerve, and PVs. Traditional RF and cryoablation techniques are associated with higher rates of complications, such as atrioesophageal fistulas, phrenic nerve palsy, and PV stenosis, leading to increased healthcare expenditures due to prolonged hospital stays and additional interventions^[3]^. Reducing complication rates with PFA improves patient outcomes and reduces the financial burden on healthcare systems by minimizing the need for costly post-procedural care and rehospitalizations^[4]^. Procedure duration and resource utilization are also important determinants of cost-effectiveness. Studies have demonstrated that PFA has a significantly shorter procedural time than RF and cryoablation, primarily due to its nonthermal, contact-independent mechanism, which enables faster lesion formation^[51]^. Shorter procedures result in lower anesthesia requirements, reduced catheter laboratory occupancy, and improved workflow efficiency, enabling more patients to be treated within the same timeframe. This increased throughput has economic implications, particularly in high-volume centers, where optimizing procedural efficiency directly translates into cost savings^[52]^. Another important aspect of cost-benefit analysis is the need for repeat ablation procedures.

**Figure 7.**
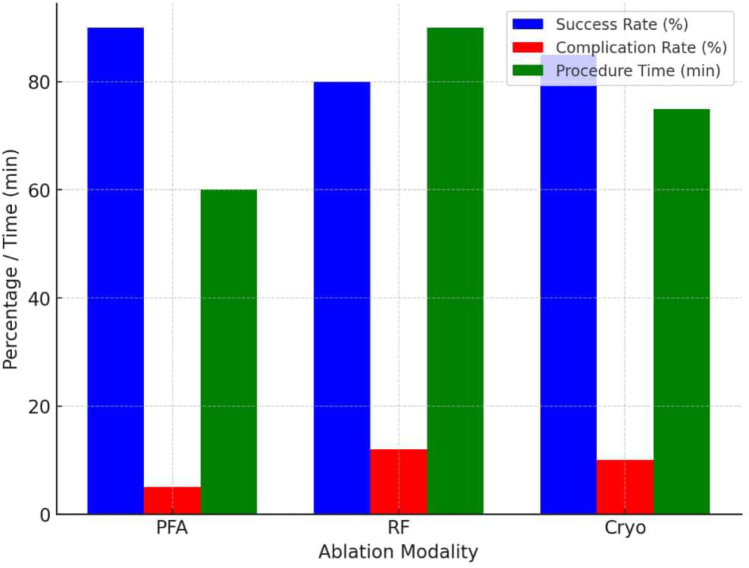
Procedural success and complication rates associated with PFA ablation are shown. The figure highlights the efficiency and safety advantages of PFA compared with conventional approaches.

AF recurrence following catheter ablation remains a significant challenge, with repeat interventions contributing to increased healthcare costs. The durability of PFA lesions has shown promise in reducing AF recurrence rates compared to traditional ablation techniques, potentially decreasing the number of patients requiring repeat procedures^[53]^. By lowering the burden of reintervention, PFA could contribute to significant long-term cost savings for healthcare providers and payers^[8]^. The economic implications of PFA also extend to hospital readmissions. Post-ablation hospitalizations due to complications such as pericarditis, heart failure exacerbation, and stroke contribute to increased healthcare costs. Studies indicate that PFA’s improved safety profile results in lower readmission rates compared to traditional methods, thereby reducing hospital resource utilization and associated expenses^[9]^. In a healthcare landscape increasingly focused on value-based care, technologies that minimize readmissions align with financial incentives to reduce overall costs while improving patient outcomes^[54]^. The adoption of PFA in different healthcare settings presents unique financial considerations. In high-income countries with advanced healthcare infrastructure, the transition to PFA may be driven by its potential for cost savings through improved procedural efficiency and reduced complication rates (see Fig. 7).

However, in resource-limited settings, the upfront costs associated with acquiring PFA technology may present a barrier to widespread implementation^[55]^. Economic evaluations in diverse healthcare environments are necessary to assess the feasibility and sustainability of integrating PFA into routine clinical practice across different regions^[12]^. As healthcare systems worldwide move towards cost-effective and patient-centered care models, the economic advantages of PFA become increasingly relevant. While more long-term data are needed to fully assess the financial impact, preliminary evidence suggests that PFA offers a viable cost-saving alternative to traditional ablation techniques. By reducing complications, optimizing procedural efficiency, and minimizing the need for repeat interventions and hospital readmissions, PFA holds promise in improving clinical and economic outcomes in managing PeAF^[56]^.

### Integration with other AF management strategies

AADs play a critical role in the periprocedural and post-ablation management of AF. Despite PFA’s ability to selectively ablate atrial tissue while preserving surrounding structures, arrhythmia recurrence remains possible, particularly in patients with advanced substrate disease. AADs, such as amiodarone, dronedarone, flecainide, and sotalol, can help maintain sinus rhythm following ablation. The EAST-AFNET 4 trial demonstrated that early rhythm control, incorporating catheter ablation and AADs, significantly reduced cardiovascular events in AF patients^[57]^. Moreover, studies indicate that short-term AAD therapy following ablation may reduce early recurrences, likely by mitigating post-procedural inflammation and promoting atrial remodeling^[58,59]^.

In addition to AADs, anticoagulation remains a fundamental component of AF management, especially in patients at risk for thromboembolism. Catheter ablation, including PFA, does not eliminate the risk of stroke, necessitating continued anticoagulation in high-risk patients. The CHA2DS2-VASc score remains the primary tool for assessing stroke risk and guiding decisions on anticoagulation. Direct oral anticoagulants (DOACs), such as apixaban, rivaroxaban, edoxaban, and dabigatran, have largely replaced vitamin K antagonists due to their superior safety profiles and ease of use^[60]^. Studies have shown that uninterrupted anticoagulation during catheter ablation reduces the risk of thromboembolic and bleeding complications, underscoring the importance of maintaining anticoagulation regardless of ablation success^[8,9]^. Beyond pharmacological therapy, lifestyle modifications and risk factor management are crucial for optimizing PFA outcomes.

Obesity, hypertension, diabetes, obstructive sleep apnea (OSA), and alcohol consumption have been strongly associated with AF development and recurrence. The LEGACY trial demonstrated that sustained weight loss in overweight and obese patients significantly reduced AF burden and improved ablation outcomes^[61]^. Similarly, the ARREST-AF study highlighted the importance of aggressive risk factor modification, showing that patients who underwent targeted interventions for hypertension, diabetes, and OSA had superior long-term ablation success^[62]^. OSA, in particular, has been recognized as a major contributor to AF pathophysiology. Studies have shown that untreated OSA is associated with increased atrial remodeling, higher recurrence rates post-ablation, and reduced efficacy of AADs. Continuous positive airway pressure therapy has been shown to improve ablation outcomes and reduce AF recurrence, emphasizing the need for routine OSA screening in AF patients undergoing PFA^[63,64]^.

Alcohol consumption is another modifiable risk factor with significant implications for AF management. The ARIC study demonstrated a dose-dependent relationship between alcohol intake and AF incidence, with even moderate consumption increasing the risk of arrhythmia^[14]^. The recently published Alcohol-AF trial showed that alcohol abstinence reduced AF burden and improved symptom control, suggesting that patients undergoing PFA should be counseled on minimizing alcohol intake^[15]^. Exercise and physical activity also affect AF management, but the relationship is complex. While moderate exercise has been associated with a reduced risk of AF, excessive endurance training can increase atrial remodeling and arrhythmia susceptibility. Studies have shown that structured exercise programs improve cardiovascular health and reduce AF burden, but individualized recommendations are necessary, particularly for athletes or highly active individuals undergoing PFA^[16]^.

Hypertension, another major risk factor, is associated with atrial enlargement, fibrosis, and an increased risk of AF recurrence after ablation. Strict blood pressure control has been shown to improve ablation success rates and reduce the recurrence of arrhythmias. The use of renin–angiotensin–aldosterone system inhibitors, such as angiotensin-converting enzyme inhibitors and angiotensin receptor blockers, has been associated with reduced atrial fibrosis and improved rhythm outcomes following catheter ablation^[17,65]^. Diabetes mellitus is another condition that negatively impacts AF outcomes. Chronic hyperglycemia promotes atrial fibrosis and electrical remodeling, thereby increasing the likelihood of recurrence after ablation. The ORBIT-AF registry demonstrated that diabetic patients have higher rates of AF recurrence and thromboembolic events despite rhythm control interventions^[19]^. Optimizing glycemic control and incorporating lifestyle modifications, including dietary changes and weight management, can enhance the efficacy of PFA and reduce the progression of AF^[20]^.

### Special populations and considerations

The elderly population represents a significant proportion of patients with PeAF, given the increased prevalence of arrhythmias with aging. Older patients often have multiple comorbidities, including hypertension, diabetes, chronic kidney disease, and heart failure, all of which can influence the safety and efficacy of ablation procedures^[1]^. PFA has been proposed as a safer alternative for this demographic due to its lower risk of collateral damage to adjacent structures, particularly the esophagus and phrenic nerve, which are areas of concern in traditional thermal ablation^[2]^. Studies have demonstrated that PFA results in reduced post-procedural complications and faster recovery times, making it an attractive option for elderly patients who may not tolerate conventional ablation techniques as well^[3]^. However, concerns remain regarding long-term durability, given that elderly patients often have more extensive atrial fibrosis, which could affect procedural success rates^[4]^.

Additionally, anticoagulation management in this population remains a crucial consideration due to the higher baseline risk of stroke and bleeding complications^[5]^. Patients with congenital heart disease or those who have undergone prior cardiac surgery present another complex subgroup for PFA. These individuals often have altered cardiac anatomy, including atrial scarring from previous surgeries, chamber enlargement, and abnormal conduction pathways, making catheter navigation and lesion durability more challenging^[6]^. Conventional RF ablation has been associated with suboptimal outcomes in this population due to difficulty achieving transmural lesions in fibrotic atrial tissue^[7]^. PFA’s nonthermal mechanism offers potential advantages by allowing more precise targeting of myocardial cells without excessive tissue destruction, thereby minimizing procedural risks such as PV stenosis or collateral damage to vascular and neural structures^[8]^. Some studies have suggested that PFA may improve success rates in patients with prior atriotomy scars, although further research is needed to determine the optimal ablation strategies in these cases^[9]^.

Additionally, long-term data on arrhythmia recurrence in this subgroup are limited, necessitating ongoing trials to assess procedural durability and outcomes^[10]^. Athletes and highly active individuals constitute another unique cohort where AF management strategies must balance rhythm control with preserving cardiovascular function. AF in athletes is often associated with heightened vagal tone and atrial remodeling secondary to long-term endurance exercise^[11]^. While catheter ablation is a preferred treatment modality in symptomatic cases, traditional RF and cryoablation techniques have raised concerns regarding potential impairment of atrial function and proarrhythmic effects^[12]^. PFA’s tissue-selective properties offer a theoretical advantage by minimizing unnecessary myocardial damage, which is particularly relevant for athletes who require optimal atrial function for performance^[13]^. Recent data suggest that PFA maintains PVI with lower arrhythmia recurrence rates in highly active individuals compared to conventional methods. However, further studies are needed to confirm these findings in larger cohorts^[14]^. Moreover, post-procedural recovery and return-to-exercise timelines may be more favorable with PFA due to reduced inflammation and procedural trauma, factors that are critical for professional and competitive athletes^[15]^.

### Regulatory approvals and guidelines

The Food and Drug Administration (FDA) plays a central role in approving novel medical devices, including PFA catheters, in the United States. In recent years, investigational device exemption studies have been conducted to assess the safety and effectiveness of PFA in treating AF. The ADVENT trial, a pivotal US study comparing PFA with traditional RF and cryoablation, has provided key insights into the performance of PFA. Preliminary results have demonstrated noninferiority and, in certain aspects, superiority of PFA in procedural safety and efficiency, thereby accelerating the FDA’s evaluation process^[66,67]^. While commercial approval for widespread clinical use is pending, early indications suggest that PFA will soon receive FDA clearance, with some catheter systems already gaining breakthrough device designation, thereby expediting the regulatory review process.

In the European Union, the European Medicines Agency (EMA) and local regulatory authorities oversee the approval of new medical devices through the Conformité Européenne (CE) marking process. The European regulatory pathway differs from the FDA’s in that it allows for earlier clinical adoption based on conformity assessment and post-market surveillance, rather than large-scale pre-market trials (see Table 4)^[68]^. PFA systems, including the Farapulse PFA system, have already received CE marking and are actively utilized across European electrophysiology centers. The European experience with PFA has been extensive, with real-world data reinforcing its safety and efficacy^[16]^. Moreover, the adoption of PFA has been particularly significant in Germany, France, and the Netherlands, where electrophysiologists have incorporated PFA into routine practice for PVI and posterior wall ablation in PeAF^[69]^. In Asia, regulatory processes vary by country. In Japan, the Pharmaceuticals and Medical Devices Agency (PMDA) has reviewed PFA systems under the Sakigake fast-track regulatory framework, recognizing their potential to improve AF ablation outcomes. The success of CE-marked devices in Europe has supported the approval process in Japan, where clinical trials have demonstrated encouraging safety profiles with reduced complications compared to conventional ablation methods^[70]^. In China, the National Medical Products Administration (NMPA) is reviewing multiple PFA systems, with an emphasis on domestic manufacturing and cost-effectiveness^[71]^.

**Table 4:** Regulatory approvals and guidelines for PFA in atrial fibrillation management, noting FDA progress toward clearance, CE-marked device use in Europe, and ongoing evaluations in Asia

Region	Regulatory body	Approval status	Clinical trials	Adoption level	Safety profile	Efficacy data	Guideline recommendations	Future updates
USA	FDA	Under review	ADVENT trial	Limited	Positive	Noninferior	Not yet included	Expected 2025
Europe	EMA	CE approved	Multiple trials	High	Positive	Favorable	Mentioned in ESC 2023	Likely expansion
Japan	PMDA	Sakigake fast-track	Ongoing trials	Moderate	Positive	Encouraging	Early mention in guidelines	More data needed
China	NMPA	Under review	Local trials	Limited	Positive	Under evaluation	Not yet included	Pending regulatory approval
South Korea	MFDS	Evaluating	Regional studies	Moderate	Positive	Favorable	Under discussion	Likely by 2026
India	CDSCO	Pending	Local studies	Limited	Under review	Limited data	Not included	Future research needed
Australia	TGA	Under assessment	Ongoing trials	Moderate	Positive	Favorable	Pending update	Expected 2025
Canada	Health Canada	Pending	Early trials	Limited	Positive	Limited data	Not included	More trials needed
Brazil	ANVISA	Reviewing	Initial studies	Low	Under evaluation	Preliminary	Not included	Long-term data required

Meanwhile, in India and South Korea, regulatory approvals are progressing, with an increasing number of clinical trials evaluating the feasibility of PFA for local populations^[72]^. Guideline recommendations for AF management continue to evolve as new evidence emerges on PFA. The 2024 American Heart Association, American College of Cardiology, and Heart Rhythm Society guidelines on AF management acknowledge PFA as a promising technology but do not yet include strong recommendations due to the need for further randomized controlled trials. However, PFA is increasingly mentioned in expert consensus statements as an alternative to RF and cryoablation, particularly for patients at high risk of complications from thermal ablation^[73]^. The European Society of Cardiology (ESC) and the European Heart Rhythm Association (EHRA) have been more proactive in incorporating PFA into their recommendations. In the 2024 ESC AF guidelines, PFA is highlighted as a viable option for PVI, particularly in patients undergoing their first ablation for PeAF^[74]^. EHRA’s consensus document further emphasizes the importance of operator experience and appropriate patient selection to maximize the benefits of PFA^[75]^.

### Study limitations

Despite the comprehensive scope of this narrative review, several limitations should be acknowledged. First, the study design is nonsystematic and inherently subject to selection bias, as article inclusion was based on the authors’ discretion and may have excluded relevant but less prominent studies. Although we employed elements of systematic methodology – such as duplicate screening, clear inclusion/exclusion criteria, and structured data extraction – this review did not include a formal meta-analysis or quantitative quality scoring of studies. Instead, we qualitatively assessed methodological quality based on study design (e.g., randomized controlled trials vs. observational cohorts), sample size, completeness of follow-up, and clarity of outcome reporting.

Second, the literature on PFA for LAPWI in PeAF remains limited, and much of the available data are derived from early feasibility studies or observational cohorts, which restrict the ability to draw definitive conclusions about long-term efficacy and safety. Third, heterogeneity exists among the included studies in terms of ablation protocols, patient populations, operator experience, and outcome definitions, which limits the generalizability of the findings. Additionally, the absence of standardized endpoints and inconsistent use of 3D mapping systems further complicates cross-study comparisons. Regarding follow-up, the included studies varied considerably, with durations ranging from 3 to 39 months. This variability, along with differences in rhythm monitoring methods (e.g., Holter monitoring, ILRs, or symptom-driven ECGs), may have influenced the reported recurrence rates and safety profiles.

Finally, the review relied exclusively on published English-language articles, which may introduce language bias and limit the inclusion of relevant non-English research.

## Future research directions and unanswered questions

The future of PFA in AF treatment includes several promising directions and unanswered questions that require further exploration. One of the most significant areas of interest is the potential for expanded indications. At the same time, PFA has demonstrated safety and efficacy for PVI and left atrial posterior wall ablation, and its application may extend to other arrhythmias. Given its superior safety profile, PFA could be investigated for treating atypical AFL, scar-related macro-reentrant tachycardias, and other atrial arrhythmias. Additionally, hybrid ablation approaches combining endocardial and epicardial PFA may enhance outcomes for patients with long-standing PeAF. This population has historically experienced lower success rates with catheter-based ablation alone^[10,11]^.

Ongoing and upcoming research aims to refine PFA technology, including the development of catheter designs optimized for uniform lesion formation and the improvement of energy delivery algorithms. Integrating real-time lesion assessment tools, such as electrogram-based monitoring and imaging modalities, could enhance procedural efficacy and reduce recurrence rates. Moreover, studies are evaluating optimal pulse waveforms, energy parameters, and lesion durability to reduce the risk of AF recurrence and proarrhythmic effects, particularly AT following posterior wall isolation^[12]^.

Several large-scale trials, including ADVENT and MANIFEST-PF, are anticipated to provide long-term data on the efficacy and durability of PFA. These studies will be instrumental in defining the role of PFA as a first-line treatment strategy for PeAF. Additionally, comparative studies evaluating PFA against RF and cryoablation regarding long-term success rates, patient-reported outcomes, and cost-effectiveness will be necessary to determine its optimal positioning in clinical practice^[13]^.

Integrating hybrid and multimodal AF treatment approaches is another key area of future research. Combining PFA with other interventional techniques, such as left atrial appendage occlusion, could lead to more thorough AF management. Additionally, robotic-assisted catheter navigation, already beneficial in RF and cryoablation, is being explored in PFA to improve precision, reduce operator dependency, and enhance reproducibility. AI-driven robotic platforms may enable fully automated PFA procedures, potentially increasing efficiency and reducing procedural variability^[14]^.

## Concluding remarks

PFA represents a transformative advancement in the treatment of PeAF, particularly for PVI and LAPWI. Its unique nonthermal mechanism offers superior tissue selectivity, reducing complications associated with traditional thermal ablation methods. Complete LAPWI with PFA demonstrates feasibility, rapidity, and safety in real-world practice, making it a viable alternative for patients with PeAF. Notably, LAPWI can be achieved even with a fluoroscopy-only approach and does not significantly extend overall procedural times.

Despite these advantages, further studies with longer follow-ups are necessary to evaluate the long-term benefits of LAPWI in patients with PeAF, particularly in terms of freedom from arrhythmic recurrences. Ongoing research should focus on refining energy delivery parameters, optimizing lesion durability, and evaluating the integration of PFA into hybrid and multimodal treatment strategies. As large-scale trials continue to provide robust evidence, PFA is poised to reshape the landscape of AF ablation, potentially expanding its indications and improving patient outcomes in the long term.

## Call to action

As PFA continues to evolve, further research is essential to optimize its application in PeAF. Clinicians and researchers should prioritize long-term studies to assess lesion durability, procedural refinements, and patient outcomes. Collaboration between electrophysiologists, biomedical engineers, and industry leaders is crucial to advancing catheter designs, refining energy delivery parameters, and integrating PFA with hybrid treatment approaches.

Regulatory bodies and guideline committees should closely monitor emerging data to update clinical recommendations accordingly. Meanwhile, healthcare institutions should facilitate access to PFA technology and training programs to ensure widespread adoption and the establishment of standardized best practices. By fostering innovation and evidence-based implementation, the medical community can fully harness PFA’s potential, ultimately improving AF management and patient care.

## Data Availability

No new data were generated or analyzed in support of this review.
